# Distinct Actin and Lipid Binding Sites in Ysc84 Are Required during Early Stages of Yeast Endocytosis

**DOI:** 10.1371/journal.pone.0136732

**Published:** 2015-08-27

**Authors:** Agnieszka N. Urbanek, Ellen G. Allwood, Adam P. Smith, Wesley I. Booth, Kathryn R. Ayscough

**Affiliations:** Department of Biomedical Science, Firth Court, University of Sheffield, Sheffield, United Kingdom; Institute of Biology Valrose, FRANCE

## Abstract

During endocytosis in *S*. *cerevisiae*, actin polymerization is proposed to provide the driving force for invagination against the effects of turgor pressure. In previous studies, Ysc84 was demonstrated to bind actin through a conserved N-terminal domain. However, full length Ysc84 could only bind actin when its C-terminal SH3 domain also bound to the yeast WASP homologue Las17. Live cell-imaging has revealed that Ysc84 localizes to endocytic sites after Las17/WASP but before other known actin binding proteins, suggesting it is likely to function at an early stage of membrane invagination. While there are homologues of Ysc84 in other organisms, including its human homologue SH3yl-1, little is known of its mode of interaction with actin or how this interaction affects actin filament dynamics. Here we identify key residues involved both in Ysc84 actin and lipid binding, and demonstrate that its actin binding activity is negatively regulated by PI(4,5)P_2_. Ysc84 mutants defective in their lipid or actin-binding interaction were characterized *in vivo*. The abilities of Ysc84 to bind Las17 through its C-terminal SH3 domain, or to actin and lipid through the N-terminal domain were all shown to be essential in order to rescue temperature sensitive growth in a strain requiring *YSC84* expression. Live cell imaging in strains with fluorescently tagged endocytic reporter proteins revealed distinct phenotypes for the mutants indicating the importance of these interactions for regulating key stages of endocytosis.

## Introduction

The actin cytoskeleton plays a central role in many dynamic cell processes including cell motility, cell organization and membrane trafficking. Actin is highly conserved and forms dynamic filaments that can be regulated through binding myriad actin-binding proteins which control its organization and turnover in response to various external and internal stimuli [1]. During endocytosis, the actin cytoskeleton is proposed to function both to provide the force required for plasma membrane invagination and subsequently during movement of the vesicle away from the plasma membrane [2, 3].

In cells, actin filament formation requires a nucleating activity to facilitate formation of an actin nucleus that then promotes the polymerization reaction. The best characterized actin nucleators are the Arp2/3 complex and formin proteins. Both of these nucleators are found in yeast, with Arp2/3 considered to provide the majority of nucleation function during actin filament formation at sites of endocytosis, while the formins are required for generation of long, unbranched filaments that are bundled to form actin cables spanning the mother-bud axis, and used as tracks for organelle movement. The Arp2/3 complex itself is not a strong nucleator of actin and requires activation by a number of proteins called nucleation promotion factors (NPFs). It is likely that these proteins ensure that actin is only polymerized at appropriate sites in the cell. The WASP family of proteins are well characterized NPFs for Arp2/3. Yeast has a single homologue of mammalian WASP called Las17, which like WASP is able to stimulate Arp2/3 activity. Deletion of *LAS17* in yeast causes disorganization of actin, and a severe inhibition of endocytosis [4–6]. However, Las17 truncations removing the Arp2/3 binding region only cause subtle defects in endocytosis. This indicated that Las17 may have Arp2/3 independent functions and most recently it was demonstrated Las17 can nucleate actin filaments independently of Arp2/3 [7]. Given that Las17 is recruited to endocytic sites 10 seconds before Arp2/3; and that mutations in a site required for Arp2/3-independent nucleation lead to strong inhibition of early stages of endocytosis, the data suggested the possibility that Las17 can initiate actin filaments at an endocytic site, which could in turn recruit Arp2/3 and type I myosins to drive the burst of actin polymerization required for invagination. However, if actin filaments are formed at this early stage it would be predicted that there are proteins required to regulate the dynamics and organization of these filaments. Spatio-temporal analysis of actin binding proteins indicates that the majority of actin binding proteins arrive at a similar or later time to Arp2/3. This includes Abp1, Sac6 the yeast fimbrin homologue, capping proteins Cap1 and Cap2, and the transgelin homologue Scp1 [8–11]. A notable exception to this is Ysc84, which arrives 2–3 seconds after Las17 but about 7 seconds before other actin binding proteins including Arp2/3 [12]. Ysc84 binds directly to actin through an N-terminal Ysc84 Actin Binding (YAB) domain, while its C-terminal SH3 domain interacts with Las17. The ability of Ysc84 to cross-link actin filaments in the absence of dimerization, led to the proposal that the YAB domain contains two actin-binding sites. Intriguingly, Ysc84 does not bind pre-formed actin filaments indicating that its association with filaments requires it to be present during filament initiation [12].

In this study we aimed to determine whether distinct actin binding sites can be defined within the N-terminal region of Ysc84, and how mutations in any sites identified would affect its ability to localize or function in vivo.

## Results

### Identification of actin binding residues within Ysc84-Nt

**Fig 1:**
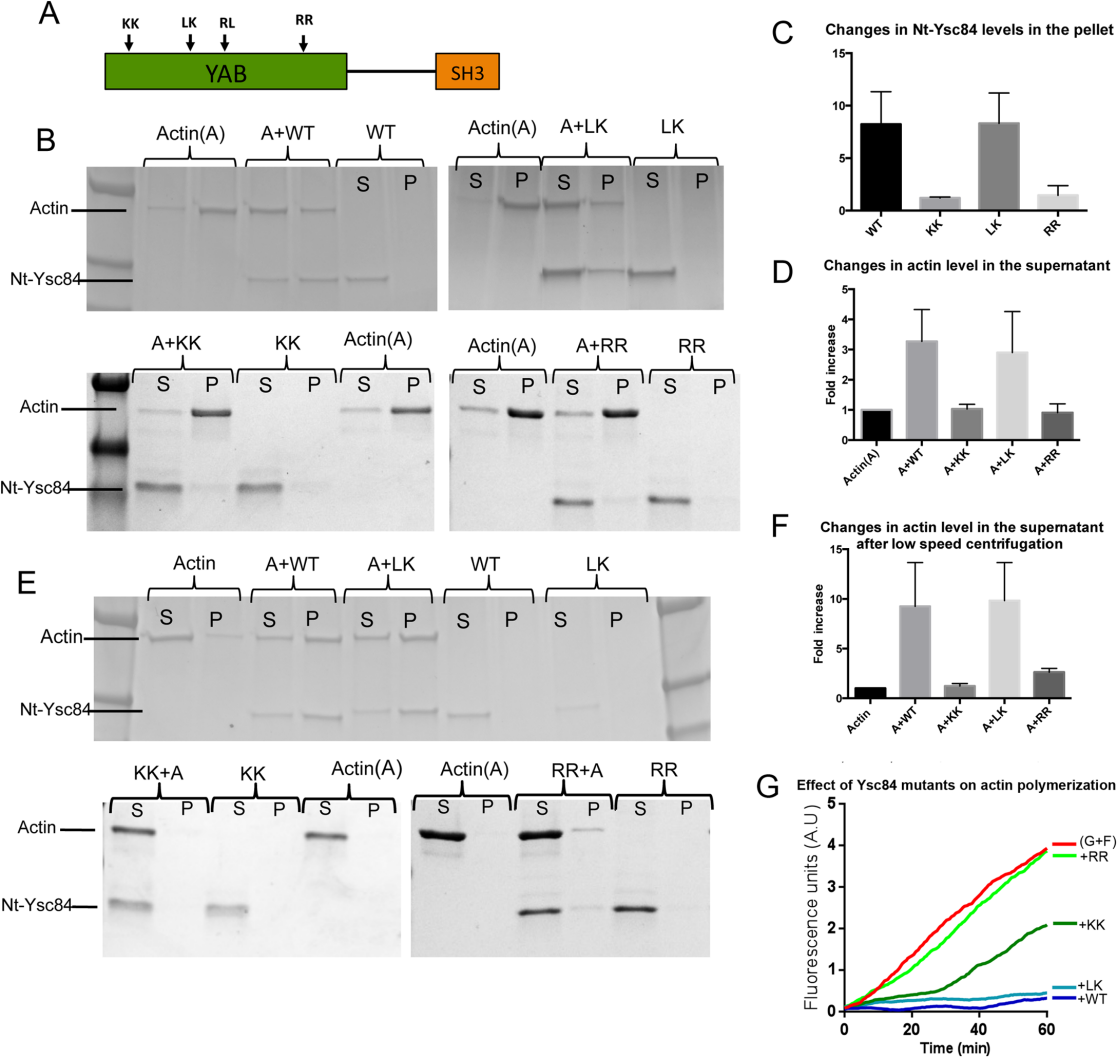
Generation of potential actin binding mutants in Ysc84 N-terminal YAB domain. (A) Schematic diagram indicating the site of four conserved pairs of residues selected for mutation. (B) Wild-type and mutant Ysc84 proteins were purified and incubated in the presence or absence of actin during polymerization. Samples were centrifuged at high speed (90K) and supernatant (S) and pellet (P) fractions separated on gels. Data from at least three independent pelleting assays were combined to determine (C) the amount of Ysc84 itself that pellets with F-actin. Error bars are standard deviation; (D) the effect of Ysc84 on the level of actin that is in the supernatant fraction. (E) Pelleting assays were also performed by spinning samples of actin and Ysc84 at low speed (15K) to assess whether the proteins were able to bundle actin filaments. Supernatant and pellet fractions were separated on gels. (F) Data from at least three independent low speed pelleting assays were combined to determine the effect of Ysc84 on the level of actin that is in the pellet fraction. (G)The effect of Ysc84 wild type and the three purified mutant proteins on interaction with actin during polymerization in the presence of actin seeds (0.5 μM G-actin + 0.5 μM F-actin) using a pyrene actin fluorimetry assay. All Ysc84 proteins were added to the reaction at 0.6 μM.

In order to investigate Ysc84 actin binding, the sequence of the N-terminal conserved YAB domain was analysed to identify possible actin binding residues. No homologies with known actin-binding motifs are identifiable, suggesting that the domain may not have simply evolved from other known actin binding proteins. Intriguingly, the full YAB domain has a high level of homology across eukaryotes, 43% identity between yeast and human, which is higher than most other actin binding proteins (*e*.*g*. comparison of yeast and human cofilin 38% identity; profilin 34% identity, capping protein 30% identity) strongly suggesting a conserved function. In other proteins, actin binding has been associated with the presence of basic amino acid residues and hydrophobic stretches incorporating leucine (L) [13], we therefore analysed the amino acid sequence of the YAB domain for conserved residues of this nature. Four amino acid pairs across the YAB domain were selected for mutation (Fig 1A; detailed sequence alignment shown in Fig. A in S1 File). The mutants: KK16,17AA, LK55,56AA, RL73,74AA and RR176,177AA were created in plasmids for yeast expression (pKA687) and in a plasmid expressing 6xHis tagged Ysc84-Nt (pKA539) using site directed mutagenesis. The mutants are referred to as KK, LK, RL and RR respectively. Based on PsiPred software (http://bioinf.cs.ucl.ac.uk/psipred/) none of the introduced mutations were predicted to disrupt the secondary structure of the protein (Fig. A in S1 File). The KK mutation is equivalent to that studied in the human homologue of Ysc84, SH3yl1, which was proposed to be part of a lipid-binding site. The RL mutant protein could not be stably expressed recombinantly from bacteria and so did not form part of the in vitro analyses but in vivo studies were carried out as described later.

Analysis of wild-type Ysc84-Nt indicates that it can interact both with F-actin (shown by the fraction of the protein pelleting following high speed centrifugation) and with either short actin multimers or G-actin monomers (shown by a shift of actin into the supernatant fraction)[12]. The observed shift into the supernatant, indicating actin monomer binding was sometimes variable, but subsequent analysis determined that incubation of Ysc84 and actin overnight at 4°C prior to high speed centrifugation gave a reproducible shift of proteins into the supernatant, compared with those left for shorter times at room temperature. Thus all pelleting assays shown were performed using these conditions.

High-speed pelleting assays were performed using the mutant Ysc84 proteins to determine whether the ability to interact with F- or G-actin was affected (Fig 1B, quantified in Fig 1C and 1D). As shown the LK mutant (Fig 1B, top gel) behaves similarly to wild-type Ysc84-Nt in that it is still able to interact with both F-actin and enter the pellet fraction, and also with monomeric/short actin filaments such that a significant proportion of actin is shifted into the supernatant. The KK and RR mutants (Fig 1B, lower gels) did not appear to bind to F-actin nor did they cause a shift of actin into the supernatant, also indicating a loss in G-actin binding. The reduction in binding to F-actin was significant as judged by unpaired students t-tests comparing to wild type samples: p value 0.0238 for KK and 0.0043 for RR mutants. The reduction in a shift of actin in the supernatant indicating monomer binding was also significant for these samples using the same analysis: p value 0.0055 for KK and 0.0005 for RR mutants.

Ysc84 has also previously been shown to bundle F-actin. Bundling can be demonstrated by pelleting of F-actin at lower speeds (15,000 rpm as described). Under these conditions actin alone does not pellet, but addition of Ysc84 to the actin induces bundling such that there is a shift to the pellet fraction (Fig 1E and 1F). Under the same conditions of centrifugation both KK and RR but not the LK Ysc84 mutant caused a reduction in actin pelleting (p value KK mutant 0.0167; RR mutant 0.0061).

A fluorescence based pyrene assay was also used to investigate the interaction of Ysc84 with actin. The fluorescence signal of pyrene actin increases during filament formation so the rate of polymerization can be monitored. Because we had previously noted that actin binding was only observed if Ysc84 was present during nucleation of filaments, we undertook an assay in which Ysc84 was incubated with a 1:1 mix of G-actin and F-actin seeds to maximize possibilities for Ysc84 interaction during the nucleation stage of polymerization. As shown (Fig 1G) the red line shows actin polymerizing in the absence of other protein. Addition of wild type Ysc84 (blue line) shows an almost total block in polymerization, indicative of sequestration or capping. A similar profile is found with the LK mutant, indicating that it is able to interact with actin to a similar extent as the wild type protein. In contrast the RR mutant (light green) shows almost no effect on the polymerization rate suggesting that the mutation prevents the actin interaction. The KK mutant shows a distinct phenotype (dark green line) suggesting a partial inhibition on actin binding at initial stages, but once overcome, polymerization proceeds at a similar rate as in the absence of the protein. Thus, as demonstrated in the pelleting assays the Ysc84 LK mutant is able to bind actin to a similar extent as wild type protein while the KK and RR mutants show reduced binding to both G- and F-actin.

In addition to the centrifugation assays, a microscale thermophoresis assay was used to determine binding affinity of Ysc84-Nt for monomeric actin. This analysis revealed a Kd of Ysc84 with G-actin of 0.85 μM. The reduced G-actin binding of the KK and RR mutants could not be reliably calculated using this method due to the concentration of our purified proteins (Fig. A in S1 File).

### Regulation of Ysc84-Nt actin binding activity is regulated by PI(4,5)P_2_ binding

**Fig 2:**
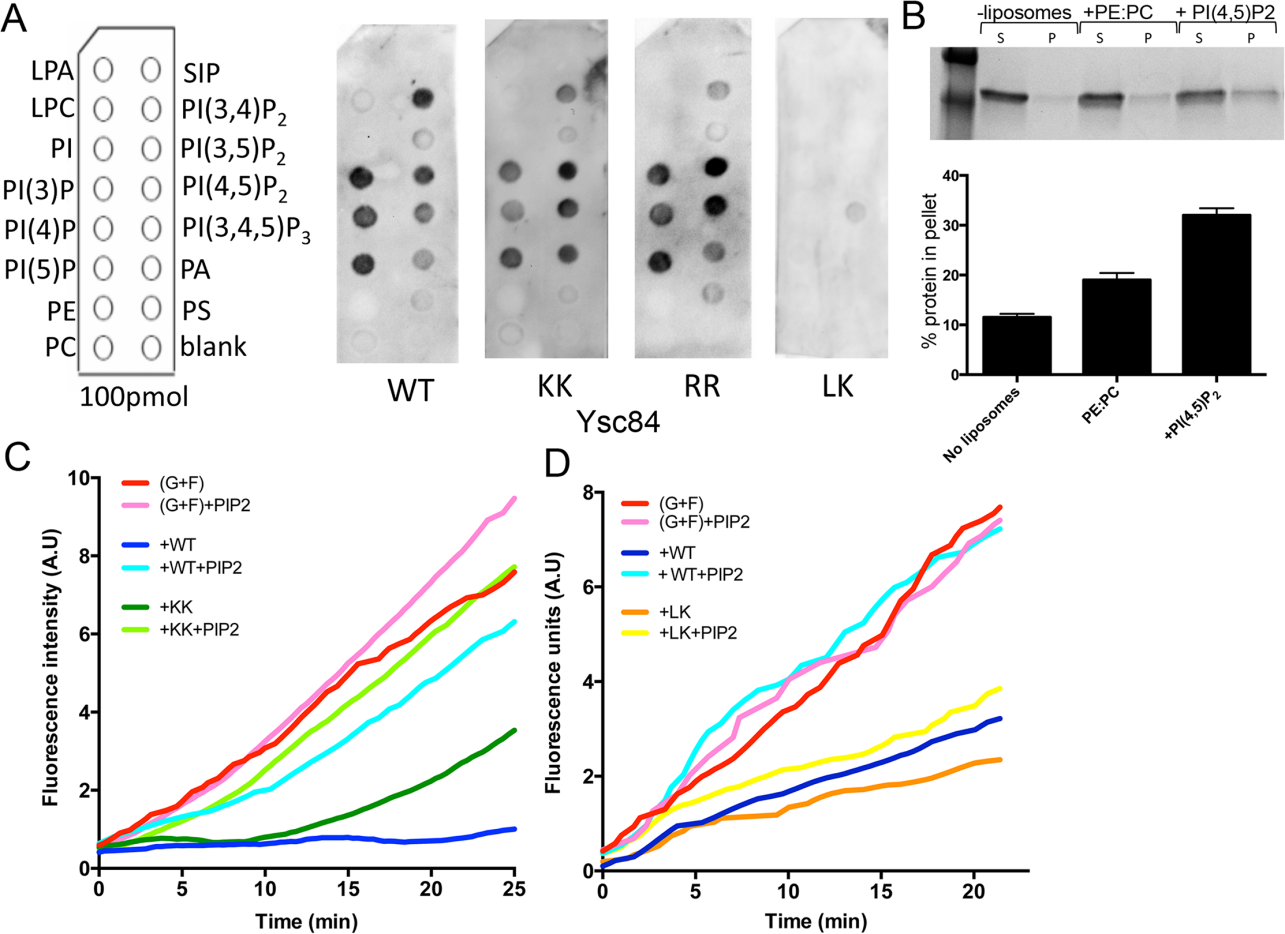
Lipid binding activity of Ysc84-Nt. (A) Dot-blot assays were performed on PIP strips to determine the specificity of Ysc84 wild-type and mutants against 15 phospholipids. The membrane was incubated with 10 μM of His-tagged purified protein and proteins detected using anti-His tag antibodies. (B) Liposome co-sedimentation assay was performed using Ysc84-Nt and 70% PE, 30% PC-based liposomes supplemented in right lanes with 10% of PI(4,5)P_2_. Proteins in the supernatant (S) and pellet (P) were visualized by Coomassie staining. Densitometry was used to determine the proportion of protein pelleting with liposomes (lower panel). Results are mean (±SD) of two independent experiments. Kinetics of F-actin barbed end elongation in the absence and presence of Ysc84 and PI(4,5)P_2_. (C,D) In a pyrene-based fluorimetry assay pre-formed actin seeds (1 μM) were mixed with 1 μM G-actin and incubated with wild type and KK (C) or wild type and LK (D) Ysc84-Nt in the absence and presence of 1.2 μM PI(4,5)P_2_.

In previous studies, it has been demonstrated that actin binding proteins can be regulated by phosphoinositides, in particular PI(4,5)P_2_ [14–18]. It was also recently reported that the N-terminal domain of the Ysc84 mammalian homologue, SH3yl1, binds to a range of phosphoinositides [19] and that lipid binding was important for the ability of SH3yl1 to function in dorsal ruffle formation in NIH-3T3 cells. To address whether Ysc84 is also able to interact with phosphoinositides, the His-tagged Ysc84-Nt domain was purified and incubated with PIP strip membranes carrying a range of lipids (Fig 2A). Binding was detected using anti-His antibodies. As shown, Ysc84-Nt binds a range of phospholipids including phosphatidyl mono- and di-phosphates. Over multiple repeats of the assay, little or no binding was observed to phosphatidylethanolamine (PE), phosphatidylcholine (PC) or phosphatidylinositol (PI). Binding to PtdIns(3,5)P_2_ was reproducibly lower than to other phosphatidyl mono- or di-phosphates. Given the localization of Ysc84 to plasma membrane endocytic sites and the known importance of PI(4,5)P_2_ regulation of endocytic events the interaction with PI(4,5)P_2_ was investigated using an alternative assay [12, 20, 21]. The Ysc84-Nt domain was incubated with liposomes containing phosphatidylcholine and phosphatidylethanolamine alone (30:70) or with liposomes also containing 10% PI(4,5)P_2_. As shown in Fig 2B, the presence of PI(4,5)P_2_ caused a statistically significant increase (p = 0.0011 using one way ANOVA test) in the amount of Ysc84-Nt able to pellet with liposomes also supporting the idea that Ysc84 is able to interact with phospoinositol lipids.

PI(4,5)P2 is known to function in multiple regulatory events at the plasma membrane including regulation of actin binding proteins [14–17, 22, 23]. PI(4,5)P_2_ also binds several endocytic adaptor proteins such as those with ENTH (epsin N-terminal homology) domains [24–27]. To address whether PI(4,5)P_2_ is able to regulate the actin binding activity of Ysc84-Nt, the pyrene fluorescence assay was performed in the presence of Ysc84 and PI(4,5)P_2_. As shown in Fig 2C and 2D, Ysc84-Nt inhibits actin polymerization (blue line) but this activity is inhibited by addition of PI(4,5)P_2_(pale blue line). Addition of PI(4,5)P_2_to actin alone does not have any inhibitory effect (pink line).

To determine whether Ysc84 mutants are affected in their phospholipid binding, PIP strip assays were performed as for wild-type protein. While the same pattern of binding as wild-type was observed for the Ysc84 KK and RR mutants, the LK mutant showed a severe impairment in all lipid binding (Fig 2A). Thus, mutagenesis of residues LK55,56 inhibits the lipid binding activity in Ysc84.

Given that the actin interacting activity of wild-type Ysc84 is severely inhibited by PI(4,5)P_2_ (Fig 2C) it was hypothesized that the actin binding of the LK mutant protein should not be subject to this same inhibition. As shown in Fig 2D, this is indeed the case with the actin sequestering activity being largely retained in the presence of PI(4,5)P_2_ (orange and yellow lines). The assay was also performed with the KK mutant that still showed a low level of actin binding at early time points (Fig 2C). As shown PI(4,5)P_2_ addition, is also able to inhibit KK actin binding indicating that this mutant, while impaired in actin binding, is still able to respond to lipid binding.

### Analysis of the effect of mutations on Ysc84-GFP localization

**Fig 3:**
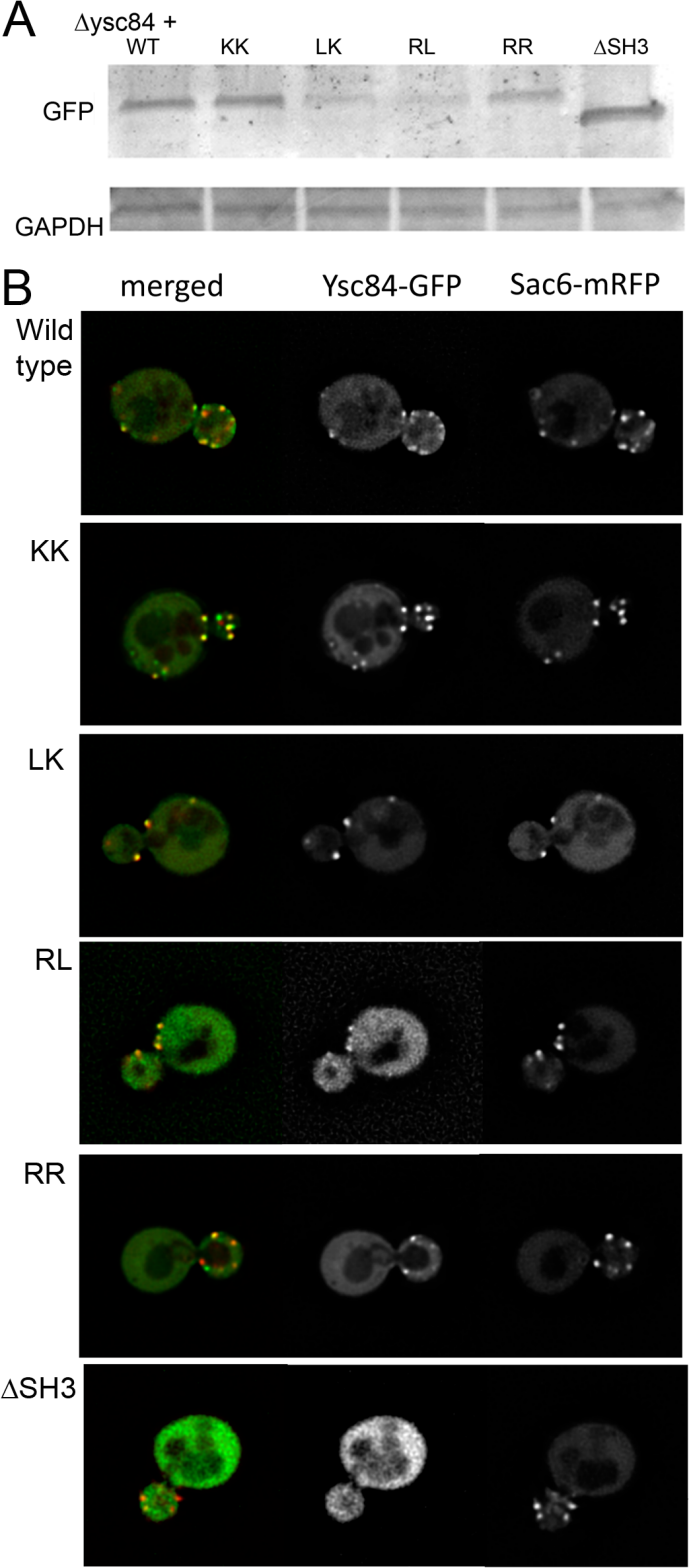
Localization of wild-type and mutant Ysc84-GFP in cells. Cells lacking *ysc84* were transformed with Ysc84-GFP WT and its mutants. (A) Levels of expression of the GFP-tagged Ysc84 protein were assessed using western blotting and anti-GFP antibodies. (B) Localization of Ysc84-GFP (wild type and mutants) and Sac6-mRFP was determined in live cells. Bar = 2 μM.

To understand how the actin and lipid binding mutations in Ysc84 affect its behaviour in cells, the KK, LK, RR and also the RL73,74AA mutations were generated in a construct expressing Ysc84-GFP. The previously reported importance of the Ysc84 SH3 domain in regulating its actin regulatory function also led us to investigate the role of this domain alongside the mutants, thus a GFP-Ysc84∆SH3 construct was generated. All mutants tagged with GFP were transformed into cells lacking endogenous *YSC84*. Transformants were grown in appropriate selective medium and imaged as live cells. As shown in Fig 3A, wild-type and mutant Ysc84-GFP were detectable in cells by western blotting. Relative to the loading control the levels of the RR and KK mutant appeared similar to wild-type, while the LK levels were slightly lower and the RL mutant which was unstable in bacteria also appeared less stable in yeast, with only a very low level detectable in cell extracts. The SH3 deletion mutant was more stable than the wild-type protein. Imaging of GFP in live cells that also expressed an actin patch marker Sac6-mRFP (Fig 3B) revealed that Ysc84-GFP co-localizes with Sac6-mRFP at the plasma membrane indicating the tagged protein is at endocytic sites. A similar localization to wild-type Ysc84-GFP was observed for the actin-binding Ysc84 mutants (KK and RR) indicating that defective actin binding does not impair recruitment to endocytic sites. In addition, the LK mutation that does not bind lipids did not prevent recruitment to endocytic sites demonstrating that lipid binding is also not necessary for localization. Deletion of the SH3 domain did however, completely inhibit localization indicating that this domain is necessary for Ysc84 to localize to endocytic sites. Although only expressed at low levels, the fourth Ysc84 mutation (RL73,74AA) was observed to localize to punctate patches at the plasma membrane. The intensity of staining was however very variable for this RL mutant with a few cells (<5%) showing staining at an intensity similar to those expressing the other mutants, while the majority of cells showed a much weaker punctate staining. This variation in expression level most likely explains the low level of Ysc84-RL mutant protein seen by blotting. Together the data support the idea that the SH3 domain, but not actin- or lipid- binding are required for Ysc84 localization to endocytic sites.

### Investigating functionality of Ysc84 mutants in a genetic background requiring *YSC84* expression

**Fig 4:**
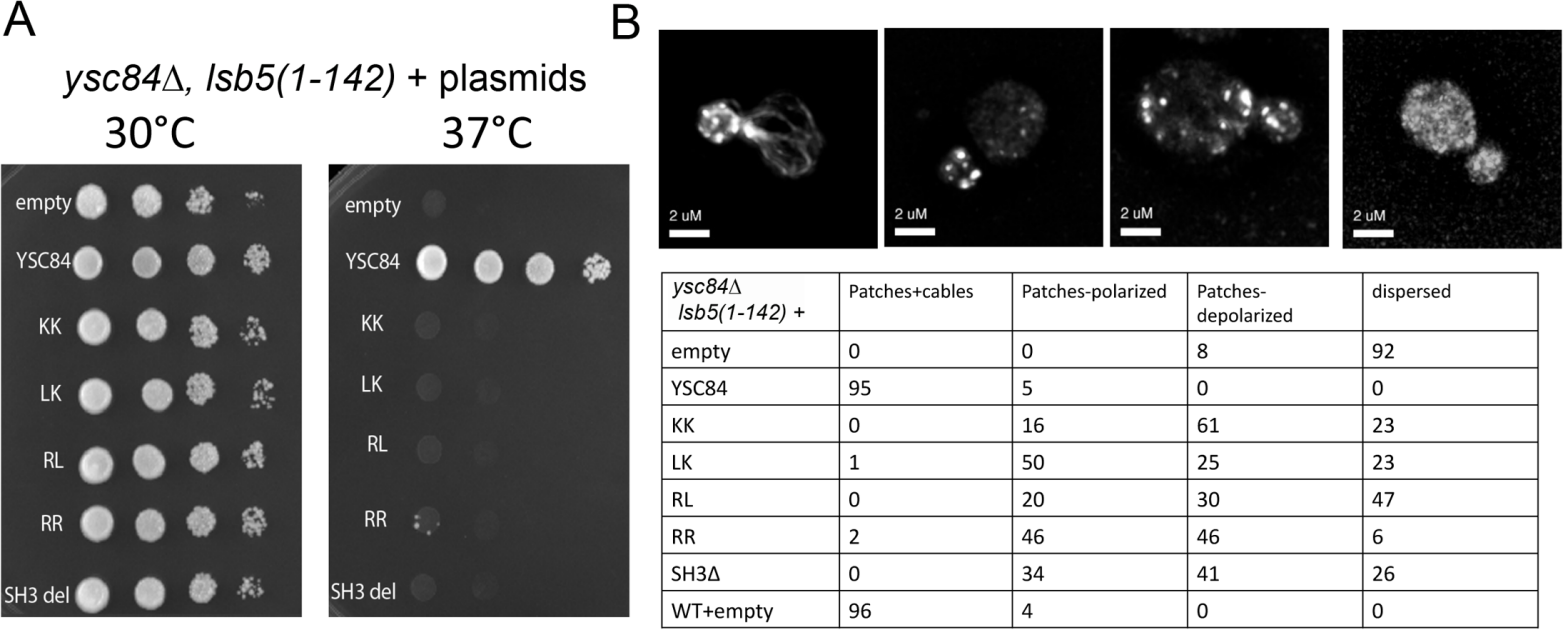
Rescue of actin and growth phenotypes of ysc84∆, lsb5(1–142) strain. *ysc84∆*, *lsb5(1–142)* strain was transformed with plasmids carrying wild-type or mutant *ysc84* or with an empty plasmid. (A) Growth on plates was assessed at 30°C and 37°C. (B) Cells were fixed and stained with rhodamine phalloidin before analysing actin organization microscopically. Actin organization in the cells was categorized as detailed.

In previous work it was shown that deletion of *YSC84* in a genetic background harbouring a truncated form of another endocytic component *lsb5* caused a severe temperature sensitive growth defect [20]. In addition, the *ysc84*∆, *lsb5*(1–142) mutant cells had depolarized and dispersed actin. To determine whether the mutant Ysc84 proteins were able to rescue these effects, *ysc84*∆, *lsb5(1–142)* cells were transformed, alongside positive and negative controls. Cells were plated at 30°C and 37°C to determine whether the mutants were able to restore Ysc84 function at elevated temperatures. As shown in Fig 4A, while re-expression of wild-type *YSC84* fully restores growth to these cells, none of the mutants restore growth despite being expressed and, in the case of most mutants, localizing to endocytic sites. The effect of the mutants on actin organization at 30°C was then analysed (Fig 4B). Four categories of actin organization were defined in cells: (1) both actin patches and cables; (2) only patches but these were largely polarized; (3) only patches but largely depolarized; (4) dispersed actin with elevated background staining and any patches were difficult to discern. In *ysc84*∆, *lsb5(1–142)* cells with re-expressed *YSC84* or in wild-type cells, ≥95% cells contained both actin patches and cables while in the *ysc84*∆, *lsb5(1–142)* cells 92% cells contained dispersed actin. The mutants showed intermediate phenotypes with the LK showing the mildest phenotype with 50% cells containing polarized actin patches. The most severe phenotype was observed with the RL mutant in which 47% cells still showed a dispersed actin phenotype, though this may well reflect the relative non-functionality and instability of this protein. Both of the actin-binding mutants (KK and RR) resulted in a large proportion of cells with depolarized actin patches, indicating partial restoration of function at 30°C.

### Effects of *YSC84* over-expression in vivo

To understand the role of the Ysc84 mutations at endocytic sites further, another assay was developed. Deletion of *YSC84* has a relatively subtle phenotype with only a slightly decreased plasma membrane lifetime of endocytic reporters, making analysis of changes difficult to quantify [12]. In order to facilitate studies, we determined whether overexpression of *YSC84* generated a more robust phenotype to allow the effect of the actin binding and lipid binding mutations to be analysed more clearly. A plasmid was generated expressing *YSC84* under the constitutive *TPI* promoter and transformed into cells expressing a range of endocytic reporter proteins. The expression level of *YSC84* was quantified by densitometry from multiple gels. The mean level of expression was 19 fold higher than that observed in wild-type cells (gel and quantitation shown in Fig. B in S1 File). The endocytic reporters investigated were Sla1-GFP (a cargo binding protein and marker of the endocytic coat), Las17-GFP (WASP homologue required for actin nucleation and activation of Arp2/3), Bbc1-GFP (proposed regulator of Las17 functioning during invagination), Myo3-GFP (type I myosin required for invagination), Sac6-RFP (fimbrin homologue, actin bundling protein) and Rvs167-GFP (amphiphysin homologue, involved in scission of endocytic vesicles). These proteins normally arrive sequentially during each endocytic cycle and can be used to determine the stage at which a defect occurs. Cells expressing the reporters were transformed either with an empty plasmid or with a plasmid overexpressing *YSC84* and grown to log phase. The lifetime of distinct GFP or RFP patches were measured from appearance to disassembly from time-lapse movies. The lifetime of at least 30 patches from 8 different cells for each strain were measured.

**Fig 5:**
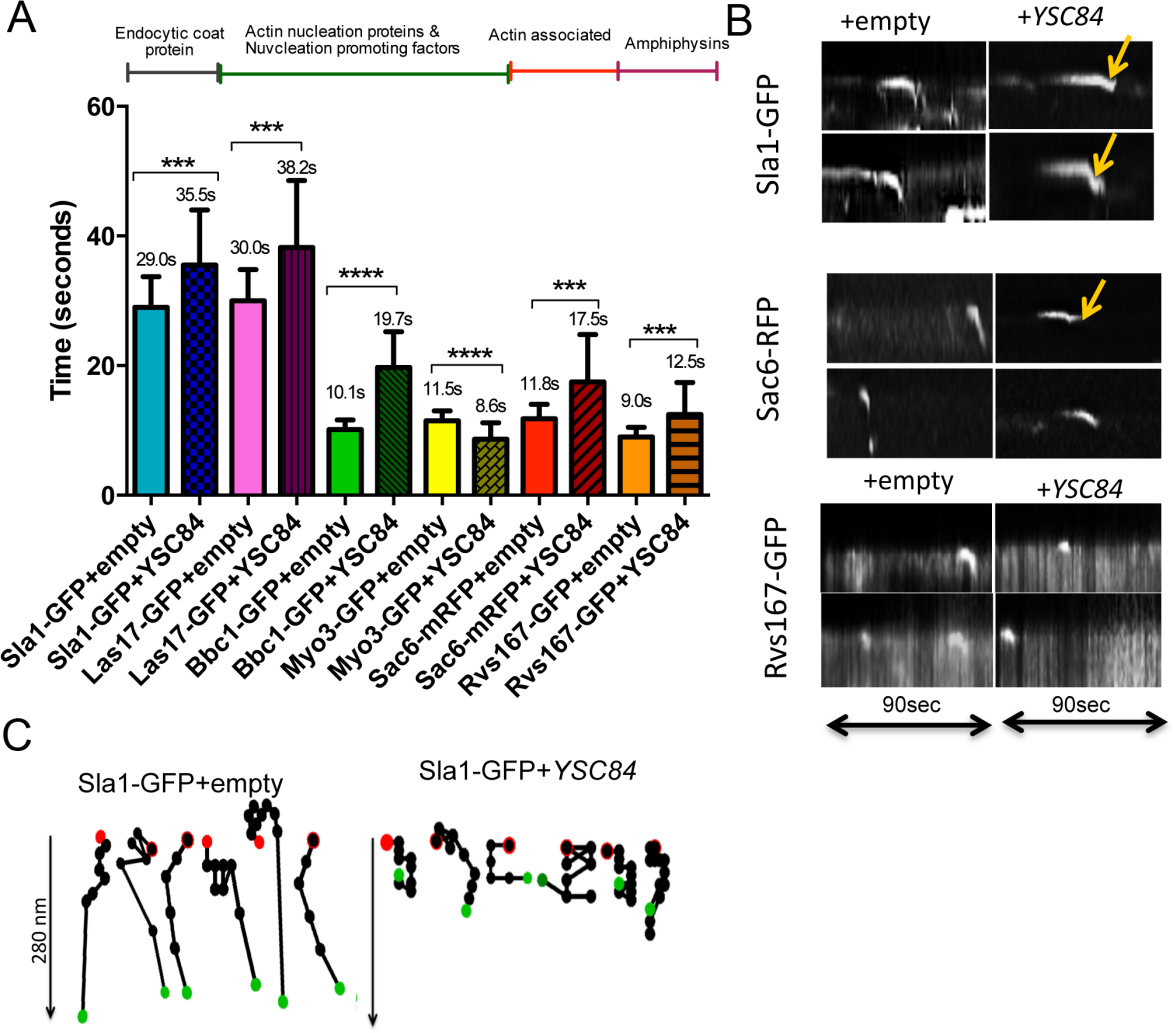
Ysc84 overexpression affects lifetime of different endocytic markers. (A) The effect of Ysc84 overexpression on endocytosis was assessed by measuring lifetime of different endocytic reporters. Movies of cells expressing fluorescently tagged different endocytic markers transformed with *YSC84* and an empty plasmid were recorded. At least 30 patches were assessed for each transformant. Error bars are SD. The differences in lifetimes of endocytic markers were calculated using two-tailed Student’s *t*-test. *** indicates a P value of 0.002 and **** ≥ 0.001. (B) Kymographs were generated from individual patches of fluorescently tagged endocytic markers expressing an empty plasmid or overexpressing Y*SC84* using the multiple kymographs ImageJ plugin. Arrows mark retractions. (C) Spot tracking of Sla1-GFP patches was performed in cells expressing an empty plasmid or overexpressing *YSC84* in manual tracking–ImageJ. Red spot–start, green spot–end. The time between spots is 1 second.

As shown in Fig 5A, the lifetimes of several markers (Sla1-GFP, Las17-GFP, Sac6-RFP, Bbc1-GFP) increased significantly when *YSC84* is overexpressed whereas, the lifetime of the later arriving proteins Myo3-GFP and Rvs167-GFP decreased. In addition, the localization of Rvs167 was altered in the *YSC84* overexpression strains with more protein localizing to the mother cell, and puncta were observed adjacent to the vacuole, suggesting displacement of Rvs167 by Ysc84 (Fig. B in S1 File).

To assess the behaviour of different proteins at the membrane further, kymographs were generated to analyse the inward movement of individual reporters (Fig 5B). The increased lifetime for Sla1-GFP and Sac6-RFP were clearly seen using this approach (Fig 5B). Furthermore, both reporters which normally show steep inward movement corresponding to invagination, showed a rate of inward movement in *YSC84* overexpressing cells about half that of wild-type cells. In addition, 38% of Sla1-GFP expressing cells and 34% of Sac6-RFP expressing cells also showed retractions of the reporters toward the membrane. Using manual patch tracking, Sla1-GFP patches for each transformant were tracked to assess patch behavior. This additional analysis of Sla1-GFP movement (Fig 5C) revealed aberrant movement in the cells overexpressing *YSC84*, highlighting the retractions back to plasma membrane seen in the kymographs (Fig 5B arrows). Overexpression of *YSC84* therefore delays both the invagination and scission stages of endocytosis.

**Fig 6:**
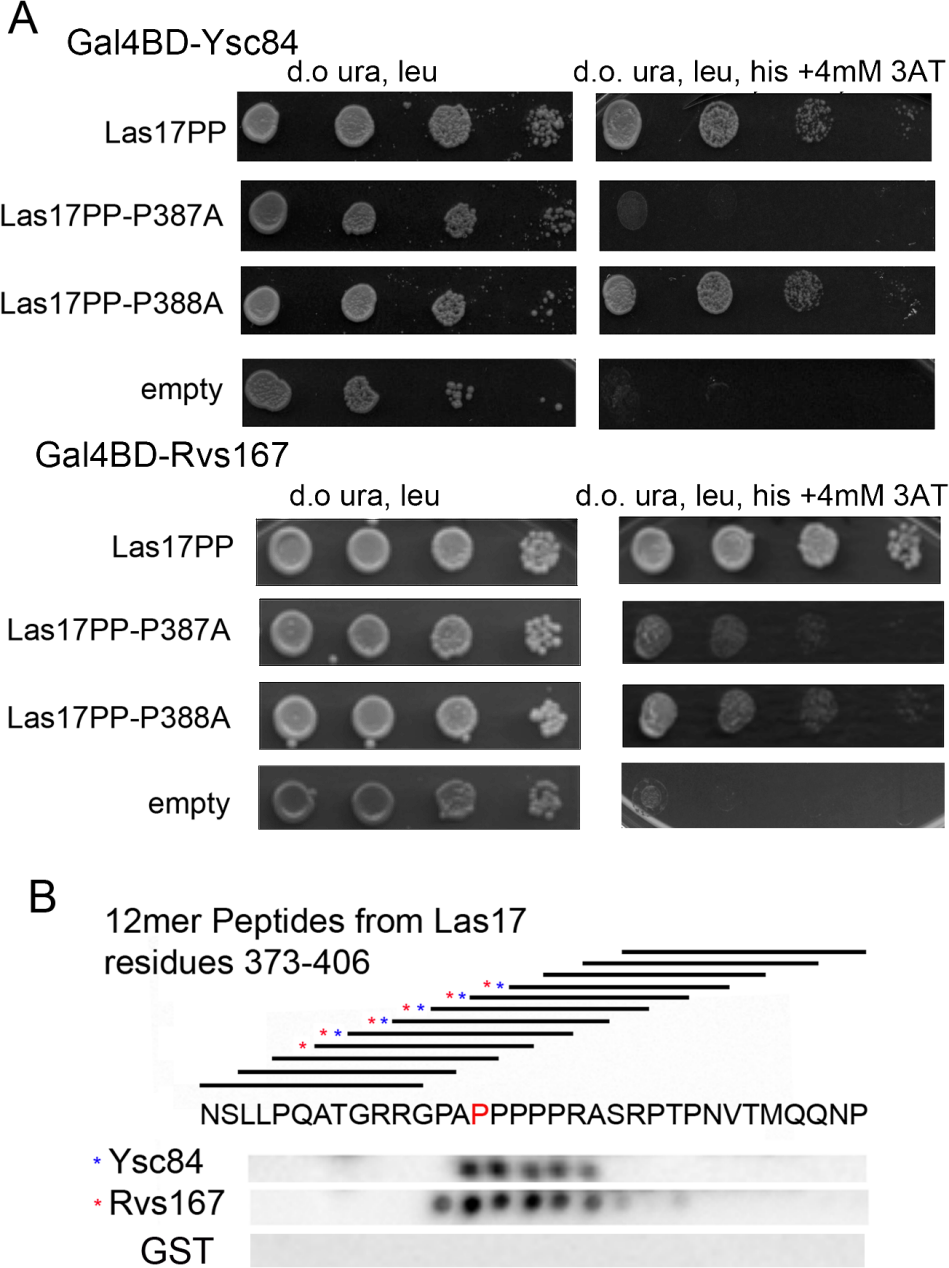
Ysc84 and Rvs167 bind to a common site on Las17. (A) Yeast two-hybrid analysis was carried out in which the polyproline domain of Las17 was carried on an activation plasmid and the Ysc84 or Rvs167 SH3 domains were expressed on the Gal4 binding domain (bait) plasmid. Mutations of Las17 carrying P387A and P388A mutations were used to reveal inhibition of binding of both SH3 domains. (B) Membrane dot blots carrying overlapping 12mer peptides of Las17 over the region 373–406 were incubated with GST fused SH3 domains of Ysc84 and Rvs167 or with GST alone. Proteins were detected by western blotting for GST.

Given the reduced lifetime of Myo3 and Rvs167 a straightforward prediction would be that excess Ysc84 may compete with Myo3 thus inhibiting invagination, and with Rvs167 reducing scission. All three proteins have been reported to bind to the yeast WASP homologue Las17 which has a pivotal role in endocytosis. To test the possibility of competitive inhibition, a yeast two-hybrid assay was performed between the Las17 polyproline region (residues 292–536) and the SH3 domains of Rvs167 and Ysc84 (Fig 6A; the Myo3-SH3 construct was self-activating and so assays were not undertaken). Both Ysc84 and Rvs167 SH3 domains clearly interact with Las17. Importantly, a mutation Las17 P387A which inhibits Ysc84-SH3 binding also significantly reduced Rvs167-SH3 binding, while an adjacent mutation Las17 P388A had little or no effect on binding either SH3 domain. The data support the idea of an overlapping binding site. To confirm that the P387 site in Las17 is able to interact directly with both Ysc84 and Rvs167 SH3 domains, and thus provide a mechanistic explanation for why Ycs84 overexpression might disrupt Rvs167 function, a SPOTS assay was used in which 12mer peptides covering the sequence 373–406 in Las17 were incubated with GST tagged SH3 domains from Rvs167 and Ysc84. Binding was detected using anti-GST antibodies. As shown in Fig 6B, there is close overlap of the series of Las17 peptides showing interaction with the two SH3 domains.

### Analysis of the effect of Ysc84 mutants on endocytosis

As shown in Fig 5, Ysc84 overexpression generated a robust phenotype, which allowed the effects of Ysc84 mutants to be analysed in cells. Yeast cells expressing the endocytic reporter Sla1-GFP and lacking *ysc84* were transformed with the *YSC84* overexpression plasmid or mutants generated in this plasmid. As noted before, with the exception of the Ysc84 RL mutant, the KK, LK and RR mutants expressed at comparable levels to the wild-type re-introduced protein. While the lack of localization of the SH3 deletion mutant might be expected to cause a phenotype similar to the *ysc84* deletion strain, the effect of inhibiting actin binding (RR, KK) or lipid binding (LK) were unknown.

**Fig 7:**
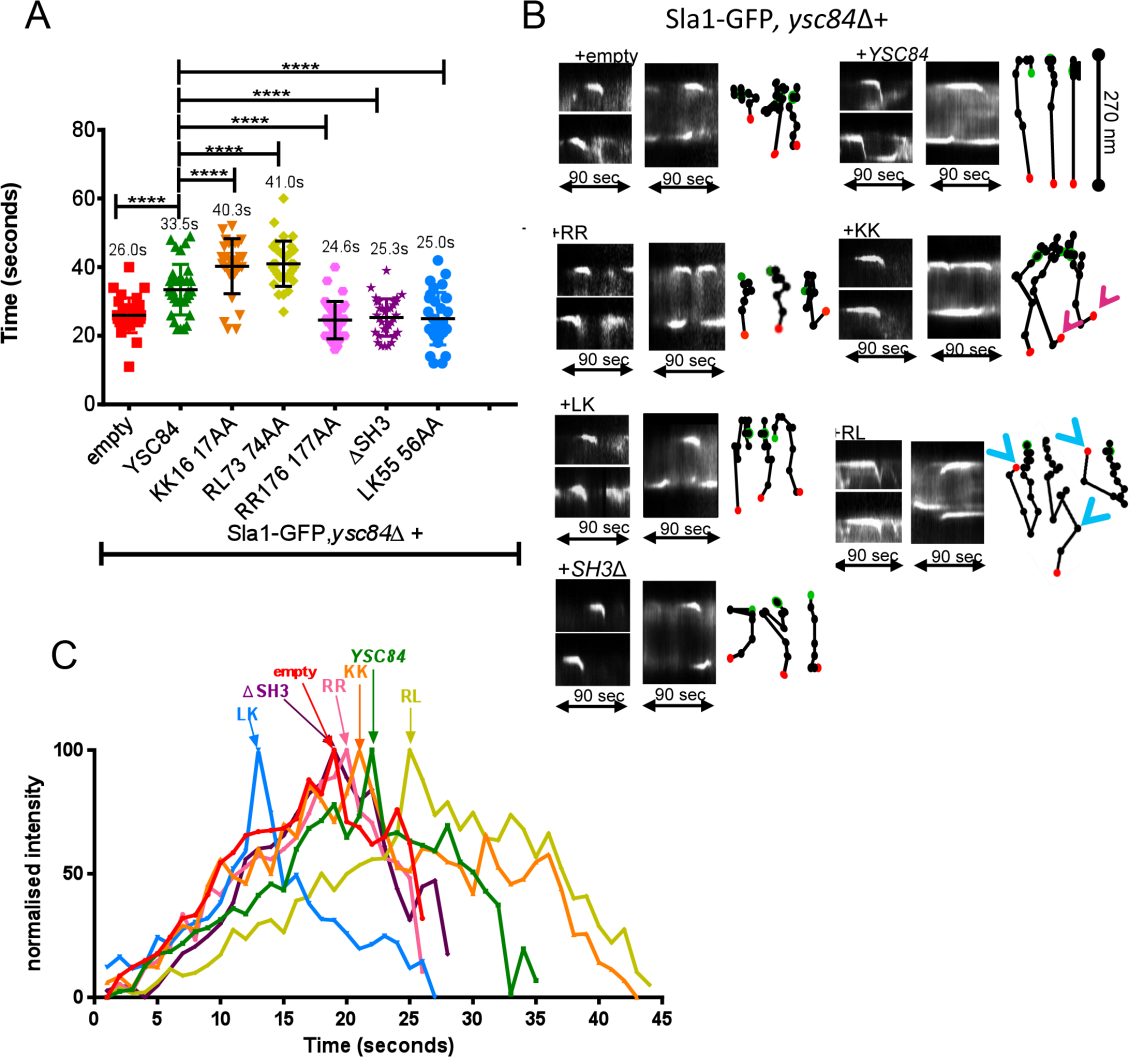
*In vivo* effects of Ysc84 mutants. (A) The effect of Ysc84 mutants on Sla1-GFP life-time was assessed by measuring patch lifetime from movies recorded of cells expressing Sla1-GFP in *ysc84* deletion strains transformed with *YSC84* and its mutants. At least 30 patches were assessed for each transformant. Error bars are SD (B) Kymographs were generated from individual Sla1-GFP endocytic patches in wild-type or Ysc84 mutant cells by using multiple kymographs ImageJ plugin. Spot tracking was created by following a movement of individual Sla1-GFP patch in ImageJ (manual tracking); *Pink arrows–*indicate small retractions of KK mutant; *Blue arrows–*retractions of RL mutant (C) Patch intensity profile. ImageJ manual tracking plugin was used to generate single patch profile intensity. An average of 4 patches were tracked for Sla1-GFP, *ysc84*Δ cells carrying empty and *YSC84*. The intensity was normalized to the same maximum.

Cells were grown in synthetic medium with appropriate supplements to exponential growth phase and visualized. As shown in Fig 7A, and previously, the Sla1-GFP lifetime is shorter in a *ysc84* null strain with an empty plasmid (26 sec) [12]. Interestingly, the mutants gave distinct phenotypes. The KK and RL mutants caused an increase in Sla1-GFP lifetime, while the RR, LK and SH3 deletion mutants cause a decreased lifetime similar to the null strain. Kymographs (Fig 7B) and intensity data (Fig 7C) reinforce these distinctions with the RR and SH3 deletion mutant giving profiles similar to the complete deletion, indicating that their mutations render them essentially non-functional despite the RR mutant being able to localize. The LK mutant also caused a reduction in lifetime of Sla1-GFP, but showed a slightly greater level of invagination suggesting restoration of some functionality, which was also indicated in the rhodamine phalloidin staining experiments. Interestingly, this mutant appears to show a peak of Sla1-GFP recruitment to patches earlier than in other strains (Fig 7C). In contrast the KK mutant has a prolonged non-motile phase before invagination and sometimes showed small retractions on invagination. This mutant shows a delay in Sla1-GFP disassembly from the endocytic site. The RL mutant, while appearing to be unstable in cells, causes some profound defects. In particular, strains expressing this mutation have a very extended Sla1-GFP lifetime and patches show protracted invagination and retraction toward the membrane. Together the mutations highlight the importance of the actin binding, lipid binding and SH3 domain interactions in facilitating the function of Ysc84 *in vivo* during endocytosis.

## Discussion

Ysc84 is able to bundle actin filaments, but as evidence for its dimerization has not been observed, it was previously proposed that there are likely to be at least two actin binding sites in the protein [12]. In this study we have identified two distinct motifs within Ysc84 that are responsible for actin binding and a separate region required for lipid binding. In the wild type protein, lipid-binding inhibits actin-binding suggesting that actin-binding of Ysc84 could be regulated by binding at the plasma membrane. We also show that the SH3 domain, but not the lipid binding or actin binding region, is required for Ysc84 recruitment to the endocytic site. All three regions are critical for Ysc84 function in vivo.

### Mapping Ysc84 actin and lipid binding sites

Mutational analysis revealed that residues RR176,177 are important for both actin monomer and filament binding, and when mutated to alanines, actin can no longer be sequestered in a form that is retained in the supernatant during centrifugation assays, nor can it sequester actin in a pyrene based assay to reduce polymerization rate. These 2 basic residues are highly conserved across eukaryotes with RR in *S*. *cerevisiae* and RK in human, mouse and chicken. In addition, these basic residues are flanked on each side by an acidic residue creating a highly charged patch available for the interaction (Fig. S1A in S1 File).

The second mutation that affected actin binding was KK16,17AA. Again both, G-actin and F-actin binding were defective as judged from actin co-sedimentation assays. Lack of F-actin binding also correlated with a lack of actin bundling in a low speed pelleting assay. Interestingly, the pyrene polymerization assay that used a combination of G-actin and F-actin seeds indicated that the Ysc84 KK16,177AA mutant did show some level of sequestration at early stages. However, once polymerization commenced, the ability to interact and reduce polymerization was lost. This assay therefore indicated a slight difference between the two actin binding mutations with the KK mutant still retaining some ability to interact with actin nuclei/seeds. A crystal structure is not available for the N-terminal YAB domain of Ysc84, and we have to date been unable to generate sufficient highly purified protein for crystallization, but further structural information would allow us to gain critical information on the relative organization of these two parts of the protein.

The other major activity of Ysc84 detected by the mutational analysis was lipid binding. Our analysis has revealed that Ysc84 is able to bind to a range of inositol phospholipids. Mutation of residues LK55,56 to alanines completely inhibits the lipid binding activity and renders the actin binding activity of this mutant insensitive to addition of lipid. The mammalian homologue of SH3yl-1 shows a high level of identity with Ysc84 and has been the subject of recent reports suggesting that it is a regulator of dorsal ruffle formation in NIH3T3 cells and that it binds SHIP2 (the PI(3,4,5)P_3_ 5-phosphatase, Src-homology 2–containing inositol-5-phosphatase 2) [19]. However, based on attempts to demonstrate binding to rabbit muscle actin, the authors suggested that SH3yl-1 is not an actin-binding protein. They did however report binding to PtdIns(3,4,5)P_3_ and various diphosphate inositol lipids containing a 5’phosphate. The mutational analysis undertaken in the report focused on the N-terminal amphipathic helix, equivalent to that containing the KK16,17 mutation studied here. They demonstrated that this helix is important for lipid binding and that a truncated protein lacking the helix is impaired in interaction with liposomes. Notably their results also indicated that about half of the lipid binding capacity lies elsewhere in the N-terminal domain. Our data would indicate that the region around IK53,54 (equivalent to LK55,56 in Ysc84) might harbour this additional functionality.

Given the high level of sequence identity of the domain, it might be expected that the actin binding function would be maintained. Evidence to support this comes from a cell-based study in which SH3yl-1 is expressed in yeast and was shown to disrupt actin organization [20]. The question arises then as to whether the lack of actin binding by recombinant SH3yl-1 is due to some inappropriate folding or lack of relevant modification of the protein when produced in *E*. *coli*. Alternatively, mammalian SH3yl-1 might only bind to cytoplasmic rather than muscle actin. Our preliminary analyses of SH3yl-1 expressed in other systems including baculovirus, indicates that actin binding capability should be further investigated. Currently, however we consider that the actin binding capacity of SH3yl-1 remains an important question.

### The importance of Ysc84 actin and lipid binding in vivo

The importance of the actin binding and lipid binding functions of Ysc84 were also investigated in vivo. First, localization of GFP-tagged Ysc84 mutants indicated that the SH3 domain, but not the actin binding nor lipid binding sites, were required for recruitment to endocytic sites. Given the in vitro properties of Ysc84 in binding Las17/WASP and actin, its overexpression would be predicted to have two major effects in cells. First, excess binding of actin monomer or potentially capping of actin nuclei might be expected to prevent appropriate filament growth required for recruitment of Arp2/3 and other actin binding proteins. Thus, a longer non-motile phase would be expected. This is observed for the reporters Sla1 and Las17 (Fig 5). Second, because Ysc84 is recruited through its SH3 domain, any other endocytic SH3 domain-containing proteins requiring the same Las17 binding site are likely to be impaired either in localization, or in their ability to remain at the endocytic site. This is supported by the observation that Myo3 and Rvs167 that also bind Las17 are reduced in their lifetime at the plasma membrane (Fig 5A). Furthermore, for Rvs167, we have used a yeast two-hybrid assay, and a direct binding approach to demonstrate overlap of the Ysc84 and Rvs167 binding site on Las17 (Fig 6).

The effect of the Ysc84 mutations was analysed using the overexpression phenotype to indicate defects in function. The RR mutant (non-actin binding); LK mutant (non-lipid binding) and ∆SH3 mutant all showed phenotypes similar to the null in terms of endocytic reporter timing suggesting that all 3 sites confer essential functions of Ysc84. Interestingly, cells expressing the LK mutant revealed an increased rate of recruitment of Sla1 at the endocytic site. We have previously demonstrated a direct interaction between Ysc84 SH3 domain and Sla1 [20], thus one possibility is that if Ysc84 is unable to interact with membrane lipids its SH3 domain is better positioned to interact with Sla1. This could also suggest that in the wild-type situation, lipid binding by Ysc84 may directly or indirectly regulate the interaction with Sla1.

Two mutants (KK and RL) caused a longer delay in invagination than observed with wild-type overexpression. The effect of the RL mutant appears to destabilise the Ysc84 protein in vitro and in vivo, so it was surprising that it had such a strong phenotype and indicates that the resulting changes in its interactions must be effective at relatively low concentrations. However, further analysis would require improved purification and stabilization conditions. The KK mutant binds lipid appropriately but has reduced G- and F-actin binding and also causes an increased patch lifetime phenotype. Given that the two actin-binding mutants (KK and RR) have distinct endocytic phenotypes despite being able to localize correctly to endocytic sites, and that the RR mutant has similar effects to the complete deletion, it suggests that KK is having a dominant negative effect. One interpretation of the data is that Ysc84 binds Las17 via its SH3 domain and aids actin polymerization by recruiting and delivering G-actin for Las17 mediated actin polymerization [7]. In the wild type situation, as an actin nucleus is generated, Ysc84 F-actin binding would then occur and this might in turn facilitate release of the monomer while leaving Ysc84 bound to the filament. The lack of G-actin binding in the RR mutant, and the lack of Las17 binding for the ∆SH3 mutant would prevent Ysc84 functioning to deliver G-actin to the forming actin nucleus or filament. The KK mutant can bind Las17 but like the RR mutant would also be defective in G-actin delivery. However our data (Fig 1G), suggests that it retains capacity to interact with actin nuclei. In the in vitro assay the conditions with high levels of proteins then allow this effect to be overcome. However, in cells it might be that the KK mutant interaction with actin nuclei is more inhibitory and reduces the overall growth rate of filaments at endocytic sites. Given that the Sla1 intensity data (Fig 7) indicates a reduced patch disassembly rate in this mutant, an additional scenario is that the KK mutation, as a consequence of continued Las17 or actin nuclei binding, prevents recruitment of a factor such as Abp1 and the Ark1/Prk1 kinases, required for turnover of actin at these sites.

In conclusion, this work identifies actin, lipid and Las17/WASP binding sites in Ysc84 Using both in vitro and in vivo approaches we have analysed the mechanistic relevance and in vivo functionality of these sites. Our work demonstrates how Ysc84 can facilitate endocytosis through coupling the key players of actin and Las17/WASP, as well as identifying a novel route of regulation mediated through lipid binding.

## Materials and Methods

### Materials

Unless stated otherwise, chemicals were obtained from Sigma-Aldrich or, Fisher Scientific. Media was from Formedium, UK (yeast extract, peptone, agar) or Sigma (minimal synthetic medium and amino acids).

### Yeast Strains, Plasmids and Cell Growth

Yeast Strains and plasmids used in this study are listed in Tables A and B in S1 File respectively. Plasmids were mutated using the QuikChange mutagenesis kit (Stratagene) according to manufacturer’s instructions. Transformations were performed using lithium acetate as described previously [28]. Cells were grown with rotary shaking at 30°C in liquid YPD medium (1% yeast extract, 2% Bacto-peptone, 2% glucose supplemented with 40 μg/ml adenine) or in synthetic medium (0.67% yeast nitrogen base and 2% glucose), with appropriate supplements. Yeast whole cell extract**s** were prepared from 5.0 OD_600_ units of yeast in liquid medium and separated by SDS PAGE (Any kD Mini-PROTEAN TGX Gel, BioRad).

### Protein purification and analysis

Rabbit skeletal muscle actin was purified and gel filtered as described previously [29]. Plasmids pKA539 (Ysc84-Nt WT), pKA812 (RL), were transformed into BL21 DE3 and pKA871 (RR), pKA915 (KK), pKA813 (LK) into C41 (Lucigen Overexpress C41(DE3) SOLOs) *E*. *coli* cells and grown to an OD_600_ 0.6. Protein expression was induced by addition of isopropyl β-d-thiogalactoside to 1 mM overnight (37°C for pKA539 (WT), pKA812 (RL), pKA813 (LK) and 30°C for pKA871 (RR), pKA915 (KK)). Induction temperatures and specific bacterial expression strains are critical for increased solubility of mutant proteins. 2 L cell pellets were resuspended in 12 ml of 1× phosphate buffer+60 mM Imidazole (10 mM Na_2_HPO_4_, 10 mM NaH_2_PO_4_, 500 mM NaCl, 60mM Imidazole pH 7.4), 200 μl protease Inhibitors (Roche, EDTA free) and the protein was then purified using HisTrap HP nickel columns (GE Healthcare) following the manufacturer's instructions. Ysc84 was dialysed into buffer (20 mM Tris, pH 8.0, 300 mM NaCl, and 0.1 mM DTT). GST-tagged proteins were purified using Sepahdex beads (GE Healthcare) following manufacturer’s instruction. Membrane PIP strips (Echelon were blocked with 5% fat free milk in 1xTBST for 2 hrs then incubated with 10 μM Ysc84 overnight at 4°C. The membrane was probed with anti-His tag antibody.

Preparation of actin seeds. Actin was polymerized by addition of 1 volume of 10 X KME (500 mM KCL, 10 mM MgCl2, 10 mM EGTA, 100mM Tris-HCl pH 8.0), to 9 volumes of actin for 10 minutes. Actin filaments were sheared by 1 minute sonication in water bath followed by 5–10 minutes incubation at room temperature.

Fluorimetry assay. 370 μl assays used actin at concentrations indicated. Pyrene-actin was added to 3%. Polymerization salts were mixed with Ysc84 and G buffer to give a final concentration of 1x KME. Polymerization was observed in a Cary Eclipse fluorimeter (emission 364 nm, slit 10 nm round; excitation 385 nm, slit 20 nm).

Actin pelleting assay. Actin and Ysc84 were pre-spun separately at 90 000 rpm (Beckman Coulter Optima Max 130K, TLA100 rotor) at 4°C for 15 minutes to remove any aggregates. Assay reagents were added in the following order: actin, G-buffer (2 mM Tris-HCl pH 8.0, 0.2 mM CaCl_2_, 1 mM NaN_3_, 0.5 mM DTT, and 0.2 mM ATP), Ysc84 and 10 X KME to 1 x. The volume of each assay was 50 μl. Assays were incubated at 4°C overnight before being spun down at 90000 rpm for 15 minutes at 4°C (for high speed assays) or at 15000 rpm (for low speed assays).

### Liposome preparation and lipid co-sedimentation assay

A mixture of phosphatidylethanolamine (PE, 70%), phosphatidylcholine (PC, 20% or 30%) and up to 10% of phosphatidyl inositol 4,5 bisphosphate (PI(4,5)P_2_) were dried under nitrogen gas and then resuspended at 1 mg/ml in 50 μl of buffer (25 mM Hepes, pH 7.5, 100 mM NaCl, 0.5 mM EDTA. Lipids were from Echelon). Lipids were then incubated for 1 hr at 60°C to allow formation of liposomes. Before mixing with the liposomes, Ysc84 was centrifuged at 100,000 rpm for 15 min at 4°C in TLA 100 rotor- Beckman to remove any aggregates. 5 μg of soluble protein was then incubated with 100 μg of liposomes for 20 min at room temperature then centrifuged at 90,000 rpm for 15 min at 20°C. Proteins that sedimented with liposomes in the pellet and unbound proteins in the supernatant were separated by SDS-PAGE followed by staining with Coomassie brilliant blue.

### Fluorescence Microscopy

Fluorescence microscopy was performed on an Olympus IX81 inverted microscope, and data deconvolved using AutoQuant software (Media Cybernetics). For live-cell imaging, cells were visualized in early log phase. Time-lapse live cell imaging of GFP-tagged proteins was performed with 1 sec time-lapse. The distance of moving fluorescence spots was measured, and arbitrary profile of intensity values, image coordinates, and tracking of patch movements were established using ImageJ. Images were exported as TIFF files and assembled using Adobe Photoshop CS2. Kymographs were assembled using ImageJ. Statistical analysis of lifetimes was performed using GraphPad Prism software.

## Supporting Information

S1 File Supplemental Materials and Methods = Text A. Supplemental references = Text B. Supplemental Table S1 = Table A. Supplemental Table S2: = Table B. Supplemental Figure S1. = Fig. A. Supplemental Figure S2. = Fig. B.
(DOC)
