# Supplementary material for: Distinct Actin and Lipid Binding Sites in Ysc84 Are Required during Early Stages of Yeast Endocytosis
Source: PLoS One. 2015 Aug 27;10(8):e0136732. doi: 10.1371/journal.pone.0136732 (PMC4552428; doi:10.1371/journal.pone.0136732)

**Supporting Information. S1 File**

**Text A. Supplemental Methods**

Yeast strains and plasmids used in this study are listed in Supplemental tables 1 and 2 respectively.

*Microscale thermophoresis* was used to determine whether the recombinantly expressed and purified Ysc84-Nt was able to interact with actin. This approach is based on the ligand binding-induced change in movement of molecules along a temperature gradient. To perform experiments with Ysc84-Nt a fluorescent label (NT-647) was covalently attached to the proteins (NHS coupling). In the MST experiment the concentration of NT-647 labeled NT-Ysc84 was constant, while the concentration of the non-labelled actin was varied between 20 μM – 1 nM. The assay was performed in G-buffer. After 15 min incubation the samples were loaded into MST NT.115 standard glass capillaries and the MST analysis was performed using the Monolith. NT.115. Concentrations on the x-axis are plotted in μM. Graph created using Prism6 software.

**Table A.** Yeast strains used in this study

| KAY | Genotype | Reference/Source |
| --- | --- | --- |
| 389 | Mat a, *ura3-52, leu2-3,112, his3Δ200, trp1-1, lys2-801* | (1) |
| 446 | Mat a, *his3Δ1, leuΔ2, met15Δ, ura3Δ* | Invitrogen |
| 513 | *Sla1-GFP:: TRP*1, *ysc84Δ:;HIS3* in KAY389 | (1) |
| 516 | Mat a, *his3Δ1, leu2Δ, ura3Δ, lys2Δ0, met15Δ, lsb5(1-142)::KanMx, ysc84::HIS* | (1) |
| 684 | Mat α,*ura3Δ0, leu2Δ0, his3Δ1, lys2Δ0, SAC6-RFP::KanMx* | (2) |
| PJ694a | Mat a, *trp1-901, leu2-3,112, ura3-52, his3Δ200, gal4Δ, gal80Δ, LYS2::GAL1_UAS_-GAL1_TATA_::HIS3,GAL2_UAS_GAL2_TATA_::ADE2, met2::GAL7-LacZ* | (3) |
| PJ69 4α | Mat α, *trp1-901, leu2-3, 112, ura3-52, his3Δ200, gal4Δ, gal80Δ, LYS2::GAL1_UAS_-GAL2_UAS_ GAL2_TATA_::ADE2* | (3) |
| 724 | Mat a*,his3Δ1, leu2Δ, ura3Δ, met15Δ, BBC1-GFP::HIS3* | Invitrogen |
| 726 | Mat a, *his3Δ1, leu2Δ, ura3Δ, met15Δ, RVS167-GFP*:*:HIS3* | Invitrogen |
| 757 | Mat a, *his3Δ1, leu2Δ, ura3Δ, met15Δ, LAS17-GFP*:*:HIS3* | Invitrogen |
| 758 | Mat a, *his3Δ1, leu2Δ, ura3Δ, met15Δ, ura3-52, MYO3-GFP::HIS3* | Invitrogen |
| 1061 | Mat a, *his3Δ1, leu2Δ, ura3Δ, met15Δ, SLA1-GFP*:*:HIS3* | Invitrogen |

**Table B.** Plasmids used in this study

| pKA | Description | Reference |
| --- | --- | --- |
| 168 | pGBDU-C1 | (3) |
| 325 | PGAD Las17 (292-536aa) | (4) |
| 526 | pEW415 pTpi - mcs URA | E. Hettema (University of Sheffield) |
| 539 | Ysc84Nt-6xHis3 (1-218aa) | (5) |
| 558 | pKA168+ Ysc84 full length | (5) |
| 687 | pKA526 pTpi + *Ysc84*(full length)*URA* | This study |
| 683 | pTEF *Ysc84-GFP* (H377R, Q424R) | This study |
| 714 | pKA325 P387A | This study |
| 715 | pKA325 P388A | This study |
| 727 | pDBT Rvs167 full length fused to BD | from A.Breton (Bordeaux) |
| 748 | GST-Ysc84 SH3 domain (411-468aa) | (Tong et al., 2002) |
| 802 | pKA687 mutated *Ysc84* RL73 73AA | This study |
| 809 | pKA687 mutated*Ysc84* ΔSH3 | This study |
| 810 | pKA687 mutated*Ysc84* KK16 17AA | This study |
| 811 | pKA687 mutated*Ysc84* LK55 56AA | This study |
| 812 | pKA539 mutated*Ysc84* RL73 74AA | This study |
| 813 | pKA539 mutated*Ysc84*  LK55 56AA | This study |
| 841 | pKA683 mutated*Ysc84-GFP* RL73 74AA | This study |
| 843 | pKA683 mutated*Ysc84-GFP* RR176, 177AA | This study |
| 849 | pKA687 mutated*Ysc84*ΔSH3 | This study |
| 871 | pKA539 mutated*Ysc84* RR176 177AA | This study |
| 915 | pKA539 mutated*Ysc84* KK16 17AA | This study |
| 930 | GST Rvs167 SH3 (424-480aa) | (6) |

**Text B References**

1. Dewar H, Warren DT, Gardiner FC, Gourlay CG, Satish N, Richardson MR, et al. Novel proteins linking the actin cytoskeleton to the endocytic machinery in Saccharomyces cerevisiae. Mol Biol Cell. 2002;13(10):3646-61.

2. Huh WK, Falvo JV, Gerke LC, Carroll AS, Howson RW, Weissman JS, et al. Global analysis of protein localization in budding yeast. Nature. 2003;425(6959):686-91.

3. James P, Halladay J, Craig EA. Genomic libraries and a host strain designed for highly efficient two-hybrid selection in yeast. Genetics. 1996;144(4):1425-36.

4. Costa R, Warren DT, Ayscough KR. Lsb5p interacts with actin regulators Sla1p and Las17p, ubiquitin and Arf3p to couple actin dynamics to membrane trafficking processes. The Biochemical journal. 2005;387(Pt 3):649-58.

5. Robertson AS, Allwood EG, Smith AP, Gardiner FC, Costa R, Winder SJ, et al. The WASP homologue Las17 activates the novel actin-regulatory activity of Ysc84 to promote endocytosis in yeast. Mol Biol Cell. 2009;20(6):1618-28.

6. Tong AHY, Drees B, Nardelli G, Bader GD, Brannetti B, Castagnoli L, et al. A combined experimental and computational strategy to define protein interaction networks for peptide recognition modules. Science. 2002;295(5553):321-4.

**Fig. A. Sequence analysis of Ysc84 and binding to actin**

(A) Multiple sequence alignment of Ysc84-Nt domain from various species. The evolutionarily conserved residues that were mutated, are indicated by arrows. (B) Predicted secondary structure of Ysc84-Nt generated using PsiPred software was used to determine whether any of the introduced mutations were likely to alter the secondary structure of Ysc84-Nt. None of the mutations were predicted to disrupt the secondary structure of the protein. (C) Binding curve for Ysc84-Nt and actin in G-buffer obtained from microscale thermophoresis experiment. Graph created in Prism6 software.


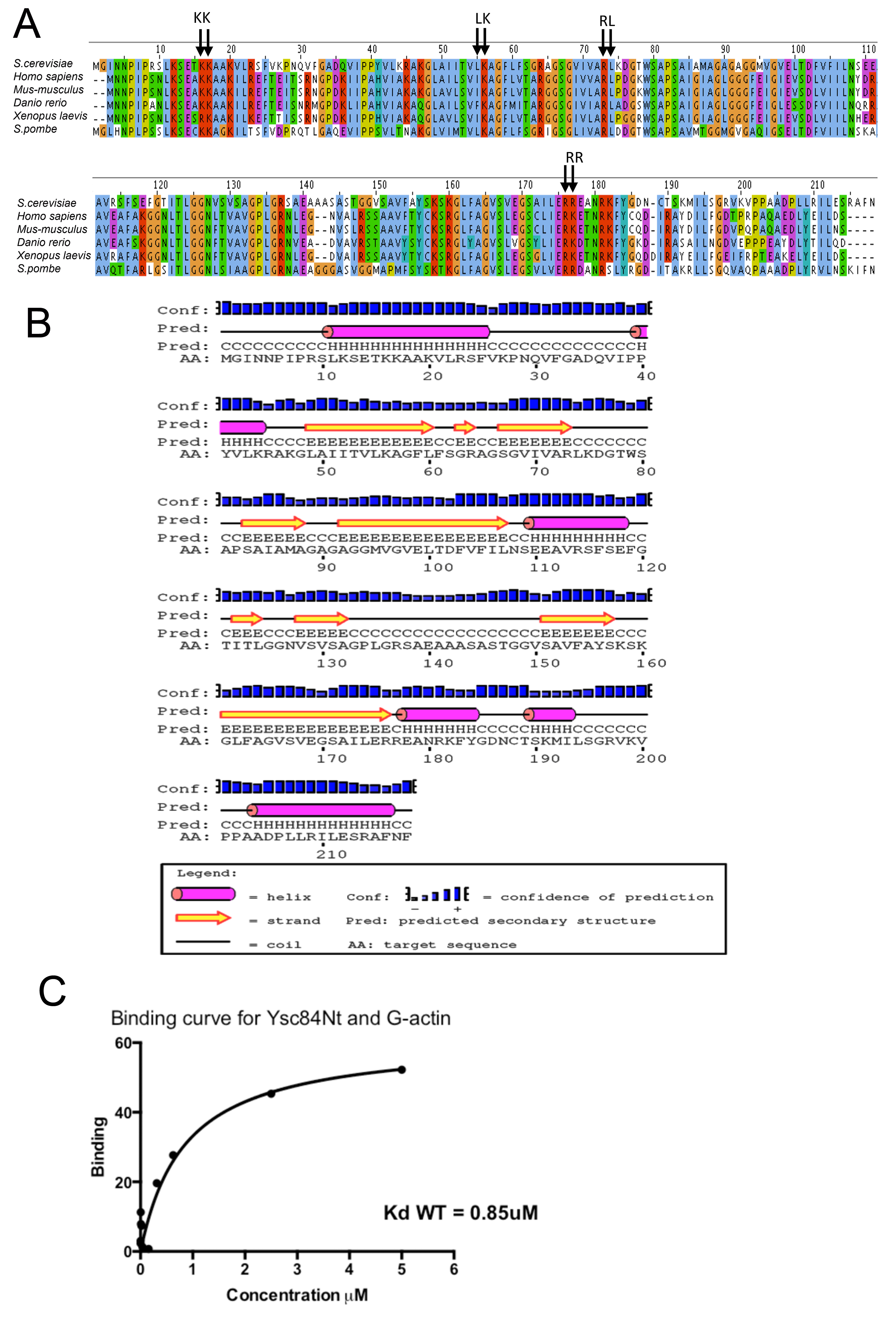


**Fig. B *YSC84* over-expression**

(A) Whole cell extracts were made from yeast strains carrying either empty vector or *YSC84* over-expressing plasmids. Expression levels of Ysc84 were judged from western blots using antibodies against Ysc84. Extracts were also probed with antibodies to GAPDH as a loading control. (B) Graph representing the fold of increase of wild-type cells carrying the *YSC84* overexpression plasmid compared to wild-type cells carrying an empty plasmid. Data was from 3 independent experiments. (C) Rvs167-GFP localization was analysed in the presence or absence of overexpressing *YSC84*. Localization of puncta of Rvs167 to the periphery of vacuoles was counted in 100 cells. 21% Wild type cells have juxta-vacuolar puncta; 46% cells overexpressing *YSC84* have Rvs167-GFP patches localizing to the vacuole periphery. (D) Whole cell extracts were made from yeast strains (wild type or *ysc84*∆) carrying either empty vector or plasmids expressing mutant versions of Ysc84. Proteins were separated on gels, transferred to PVDF membranes and probed with antibodies against Ysc84. Lower part of the gel was stained with coomassie dye to assess loading levels.


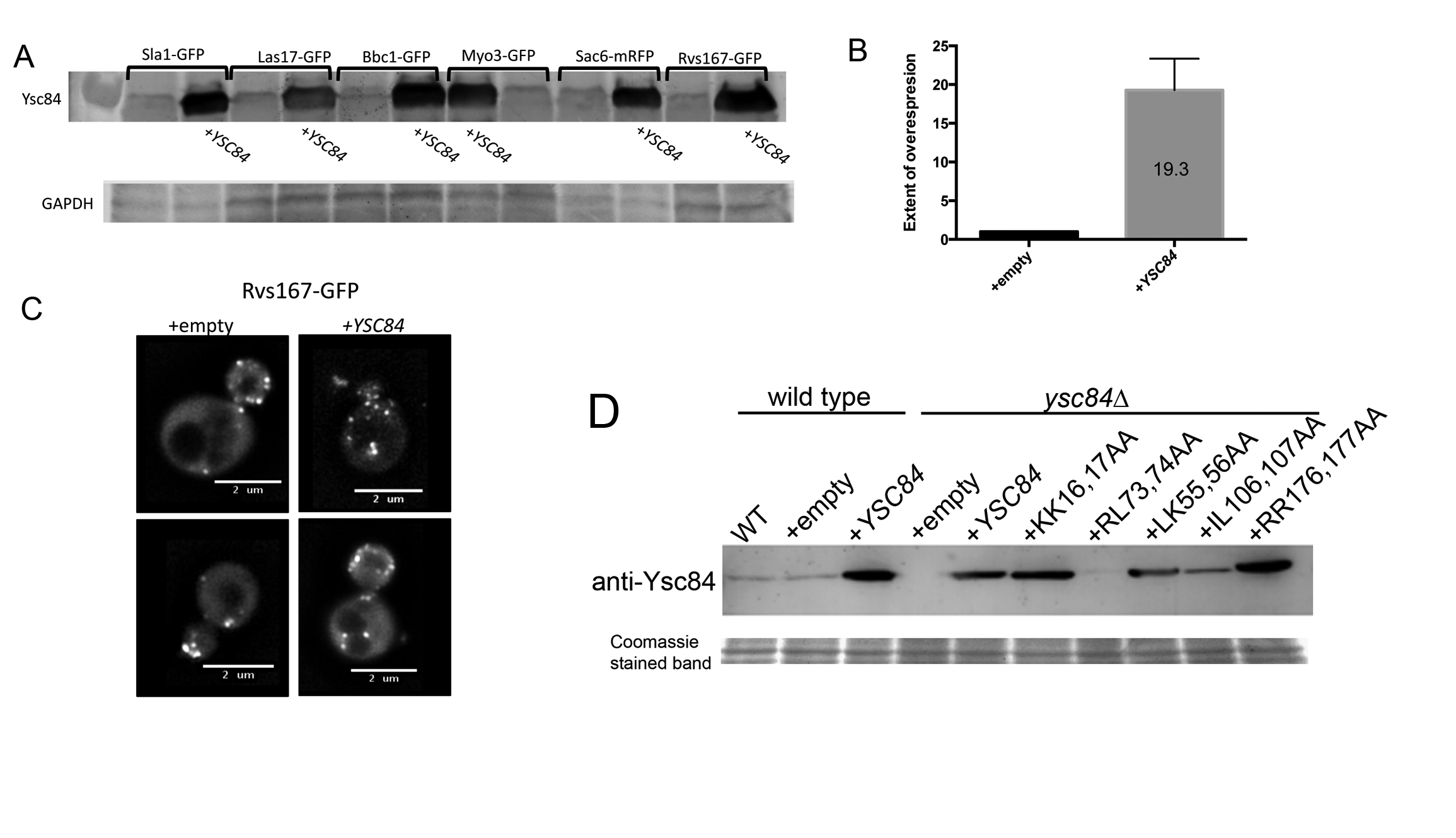

Supplement: S1 File — Supplemental references = Text B. Supplemental Table S1 = Table A. Supplemental Table S2: = Table B. Supplemental Figure S1. = Fig. A. Supplemental Figure S2. = Fig. B. (DOC) [file pone.0136732.s001.doc]
